# D-Alanine-Controlled Transient Intestinal Mono-Colonization with Non-Laboratory-Adapted Commensal *E*. *coli* Strain HS

**DOI:** 10.1371/journal.pone.0151872

**Published:** 2016-03-22

**Authors:** Miguelangel Cuenca, Simona P. Pfister, Stefanie Buschor, Firuza Bayramova, Sara B. Hernandez, Felipe Cava, Erkin Kuru, Michael S. Van Nieuwenhze, Yves V. Brun, Fernanda M. Coelho, Siegfried Hapfelmeier

**Affiliations:** 1 Institute for Infectious Diseases, University of Bern, Bern, Switzerland; 2 Graduate School GCB, University of Bern, Bern, Switzerland; 3 Laboratory for Molecular Infection Medicine Sweden, Department of Molecular Biology, Umeå Centre for Microbial Research, Umeå University, Umeå, Sweden; 4 Department of Chemistry, Indiana University, Bloomington, Indiana, United States of America; 5 Department of Biology, Indiana University, Bloomington, Indiana, United States of America; University of Osnabrueck, GERMANY

## Abstract

Soon after birth the mammalian gut microbiota forms a permanent and collectively highly resilient consortium. There is currently no robust method for re-deriving an already microbially colonized individual again-germ-free. We previously developed the *in vivo* growth-incompetent *E*. *coli* K-12 strain HA107 that is auxotrophic for the peptidoglycan components D-alanine (D-Ala) and *meso*-diaminopimelic acid (Dap) and can be used to transiently associate germ-free animals with live bacteria, without permanent loss of germ-free status. Here we describe the translation of this experimental model from the laboratory-adapted *E*. *coli* K-12 prototype to the better gut-adapted commensal strain *E*. *coli* HS. In this genetic background it was necessary to complete the D-Ala auxotrophy phenotype by additional knockout of the hypothetical third alanine racemase *metC*. Cells of the resulting fully auxotrophic strain assembled a peptidoglycan cell wall of normal composition, as long as provided with D-Ala and Dap in the medium, but could not proliferate a single time after D-Ala/Dap removal. Yet, unsupplemented bacteria remained active and were able to complete their cell cycle with fully sustained motility until immediately before autolytic death. Also *in vivo*, the transiently colonizing bacteria retained their ability to stimulate a live-bacteria-specific intestinal Immunoglobulin (Ig)A response. Full D-Ala auxotrophy enabled rapid recovery to again-germ-free status. *E*. *coli* HS has emerged from human studies and genomic analyses as a paradigm of benign intestinal commensal *E*. *coli* strains. Its reversibly colonizing derivative may provide a versatile research tool for mucosal bacterial conditioning or compound delivery without permanent colonization.

## Introduction

The mammalian microbiota influences the biology of its host at many levels. As a consequence, a large number of human conditions are not only shaped by the host’s genetic predisposition, external environment and diet, but also the microbiota composition. However, the high microbiota variability between individuals and between different experimental vivaria (often synonymously referred to as “hygiene status”) generates a growing demand for new and improved animal models that provide better experimental control over microbiota composition. Numerous studies, spanning many decades, have utilized axenic/ germ-free animals [1] and gnotobiotic animal models with simplified defined microbial compositions [2,3] to greatly advance our current understanding of host-microbial interactions. Comparing host phenotypes in complete or selective absence and presence of microbes can be highly informative. Manipulating simple microbiotas by experimentally increasing the complexity with new immigrants is generally technically easier than permanently eliminating members of an established consortia. Although antibiotic treatments provide a means for the reduction of density and complexity of an already established microbiota, it is incomplete and unsustainable without continued antibiotic administration [4] and can lead to blooms of unsusceptible or resistant microbes. Also the recovery from the antibiotic treatment back to the original state is often incomplete and irreproducible [5], potentially causing persistent dysbiosis.

We recently developed a reversible live microbial colonization model that allowed the fully transient intestinal association of germ-free animals with a live commensal bacterium, the *in vivo* auxotrophic commensal *E*. *coli* strain K-12 mutant HA107 (relevant genotype: Δ*alr* Δ*dadX*Δ*asd*). This mutant strain strictly depends on external supplementation with the bacteria-specific amino acids D-alanine (D-Ala) and *meso*-diaminopimelic acid (Dap) for growth. Both compounds are essential bacterial cell wall (= peptidoglycan) components without which muropeptide crosslinks between peptidoglycan polymers cannot be formed. Unless supplemented with both compounds, these bacteria cannot synthesize a rigid cell wall and fail to proliferate. Unlike the standard L-amino acids, host metabolism and diet cannot supply intestinal *E*. *coli* HA107 with these these two necessary bacteria-specific amino acids, allowing the quantitative and fully transient controlled association of germ-free animals with (*in vitro*-grown) live microbes followed by the rapid recovery to again-germ-free status [6]. This reversible colonization model has since been successfully used to study the dynamics of intestinal microbiota-induced immunity and disease [6–9].

Although commensal *E*. *coli* represents a highly relevant early colonizer of the human gut [10] and includes strains with probiotic potential (e.g. *E*. *coli* Nissle 1920; [11]), the rather lab-adapted K-12 strain is not the most biologically representative *E*. *coli* strain. Its rough phenotype alone (repeated *in vitro* passaging over decades led to loss-of-function of O-antigen biosynthesis due to a spontaneous mutation), among numerous other mutations, have decreased its intestinal fitness [12].

To allow studies of reversible commensal *E*. *coli* colonization in a more representative bacterial genetic background we therefore re-constructed the genotype of K-12 strain HA107 in the well-characterized, smooth (complete LPS O-antigen structure), better colonizing, and human-trial-tested benign human commensal strain *E*. *coli* HS [13,14] by introducing genomic deletions of the genes *alr*, *dadX* and *asd*. Here, we describe the necessary genetic optimizations required in this bacterial genetic background and the improved phenotype of the new bacterial strain *in vitro* and *in vivo*. This improved transient *E*. *coli* colonization model may be further extended in similar form to other microbial species and utilized for probing a multitude of host responses to bacterial inoculation, or as vector for bacterial metabolite and protein delivery without permanent colonization of the host.

## Results

### Genetic engineering of fully D-Ala- and Dap-auxotrophic *E*. *coli* HS

**Fig 1:**
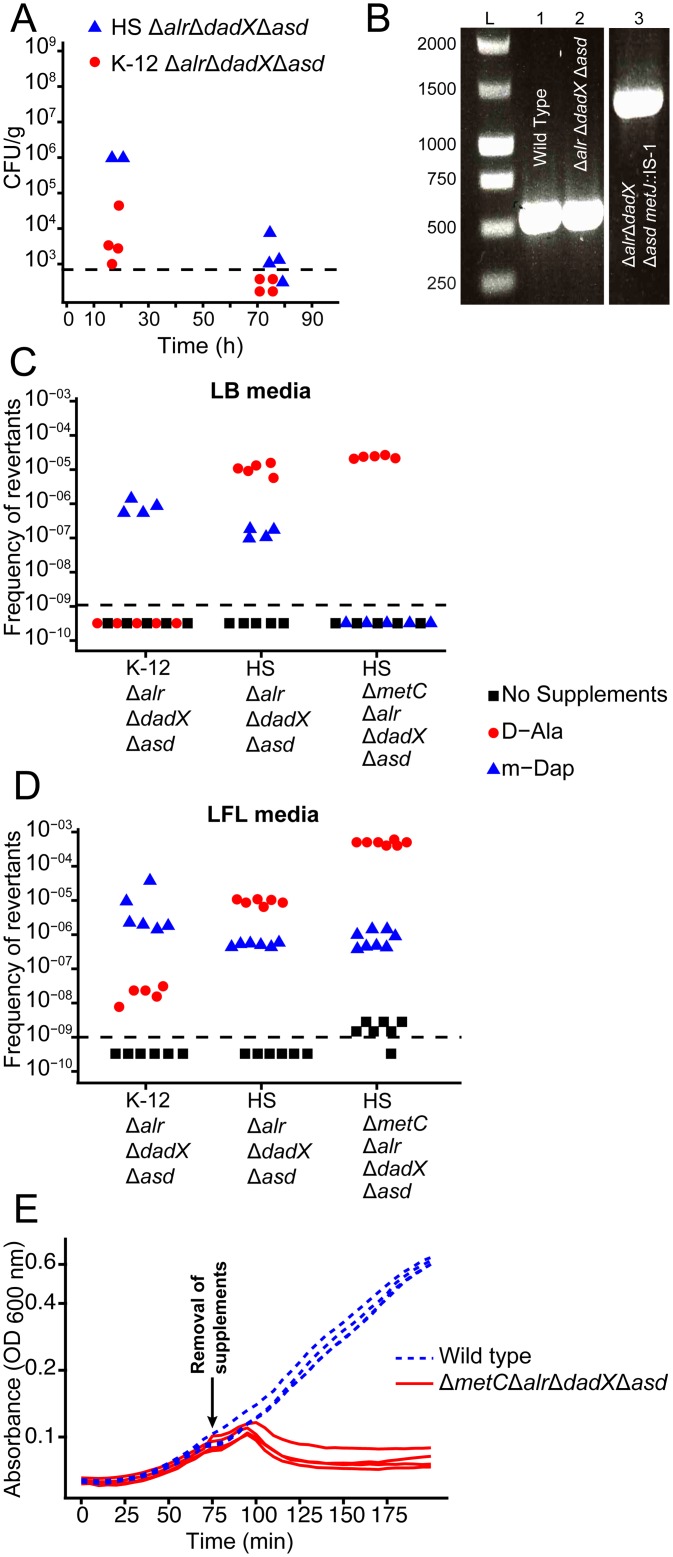
Phenotypic characterization of auxotrophic *E*. *coli* HS mutants. **(A)** Fecal bacterial loads from mice that had been gavaged with 10^10^ CFU of *E*. *coli* HS Δ*metC* Δ*alr* Δ*dadX* Δ*asd* (filled blue triangles or *E*. *coli* K-12 Δ*alr* Δ*dadX* Δ*asd* (strain HA107; filled red circles), each symbol represents one individual. **(B)** Insertion sequence (IS-)1 insertion observed in HS Δ*alr* Δ*dadX* Δ*asd* after *in vitro* selection for D-Ala auxotrophy reversion. PCR amplification of the genomic region of *metJ* from *E*. *coli* HS wild type (lane 1), *E*. *coli* HS Δ*alr* Δ*dadX* Δ*asd* original stock (lane 2), and *E*. *coli* HS Δ*alr* Δ*dadX* Δ*asd* D-Ala^+^ revertant selected on LB + Dap (lane 3) reveals a mobile genetic element insertion in the *metJ* ORF that was identified by sequencing as IS-1. **(C, D)** Frequency of auxotrophy revertants in K-12 Δ*alr* Δ*dadX* Δ*asd*, HS Δ*alr* Δ*dadX* Δ*asd* and HS Δ*metC* Δ*alr* Δ*dadX* Δ*asd* selected on LB **(C)** or LFL **(D)** containing Dap (blue triangles, D-Ala^aux^ revertants), D-Ala (red circles, Dap^aux^ revertants), or no supplements (black squares; D-Ala^aux^ Dap^aux^ double-revertants. **(E)** Bacterial growth curves of *E*. *coli* HS Δ*metC* Δ*alr* Δ*dadX* Δ*asd* (solid red line) and wild type (dotted blue line) before and after removal of D-Ala and Dap from the media (arrow indicates time point of removal).

We reconstructed the genotype of the reversible intestinal colonization prototype strain *E*. *coli* K-12 HA107 [6] in the genetic background of the less laboratory-adapted commensal *E*. *coli* strain HS [13,14] by deleting the genes *alr* (alanine racemase-1), *dadX* (alanine racemase-2) *and asd* (aspartatesemialdehyde dehydrogenase). We characterized the resulting D-Ala- and Dap-auxotrophic (D-Ala^aux^ Dap^aux^) strain HS Δ*alr* Δ*dadX* Δ*asd* in an *in vivo* pilot experiment by quantifying fecal bacterial shedding from germ-free mice following gavage with approximately 4x10^10^ bacteria. The intestinal bacterial clearance of HS Δ*alr* Δ*dadX* Δ*asd* was delayed compared to *E*. *coli* K-12 HA107 (Fig 1A), suggesting a leaky phenotype in this strain background. Others have recently demonstrated that an *alr dadX* double mutation in *E*. *coli* K-12 confers D-Ala auxotrophy only in methionine-rich complex media, but not in methionine-limited minimal media. Moreover, the abolition of methionine repression of the putative alanine racemase *metC* by mutation of the repressor *metJ* confers D-Ala-independent growth of *alr dadX* mutants in all media [15]. We found that whilst D-Ala auxotrophy revertants with a spontaneous Insertion Element (IS)-1-mediated disruption of *metJ* could readily be selected *in vitro* (Fig 1B), intestinal *ex vivo* re-isolates of *E*. *coli* HS Δ*alr* Δ*dadX* Δ*asd* remained D-Ala-dependent on rich media (8 clones isolated up to 5 days post gavage were tested).

Thus, the intestinal habitat does not appear to select for D-Ala^aux^ revertants but may be permissive for residual growth of *alr dadX asd* mutants due to methionine limitation, leading to an *in vivo* colonization phenotype solely limited by Dap auxotrophy that we previously found to be leaky [6]. This hypothesis was corroborated by the finding that spontaneous Dap-independent mutants, selected on LB + D-Ala medium, were much more frequent in the HS (mean frequency = 1.5x10^-5^) than in the K-12 (frequency < 1x10^-9^) background (Fig 1C, red symbols). To block this escape route, we therefore additionally deleted *metC*, generating the fully D-Ala auxotrophic quadruple mutant HS Δ*metC* Δ*alr* Δ*dadX* Δ*asd* (D-Ala^full-aux^ Dap^aux^). Further selection experiments on LB + Dap media confirmed a marked decrease of D-Ala reversion frequency of HS Δ*metC* Δ*alr* Δ*dadX* Δ*asd* compared to HS Δ*alr* Δ*dadX* Δ*asd* and K-12 Δ*alr* Δ*dadX* Δ*asd* (Fig 1C, blue symbols). Thus, in the HS strain background D-Ala auxotrophy confers markedly more effective growth control than Dap auxotrophy. Since the reversion rates on LB media were below the detection limit for many conditions, we additionally employed a more advanced culture method with a better recovery rate for bacterial strains with instable cell wall structure: L-form-like (LFL) media [16], an osmoprotective sucrose- and Mg^2+^-supplemented rich medium. On LFL, D-Ala^aux^ revertants could be selected at roughly 10-fold higher frequencies than on LB media (Fig 1D, blue symbols), with a no longer significant difference between HS Δ*metC* Δ*alr* Δ*dadX* Δ*asd* and HS Δ*alr* Δ*dadX* Δ*asd*, indicative of a *metC*-independent mechanism. Moreover, on LFL medium we observed an increased frequency of slow-growing Dap revertants of HS Δ*metC* Δ*alr* Δ*dadX* Δ*asd* (Fig 1D; compare red symbols in panels C and D). These clones are reminiscent of an older report that showed that *metC* mutation in Dap-auxotrophic *E*. *coli* can lead to the over-accumulation and incorporation of the Dap homologues *meso*-lanthionine and L-*allo*-cystathionine instead of Dap into the cell wall [17]. However, also under the highly permissive stabilizing conditions in LFL medium, D-Ala and Dap auxotrophies combined acted synergistically to reduce double-reversion frequency to very low levels (Fig 1D, black symbols). To allow the detection of such revertants also *ex vivo*, LFL media (unsupplemented and D-Ala/ Dap supplemented) were also used for all following animal experiments.

### Normal cell wall biochemistry of *in-vitro* grown auxotrophs

**Fig 2:**
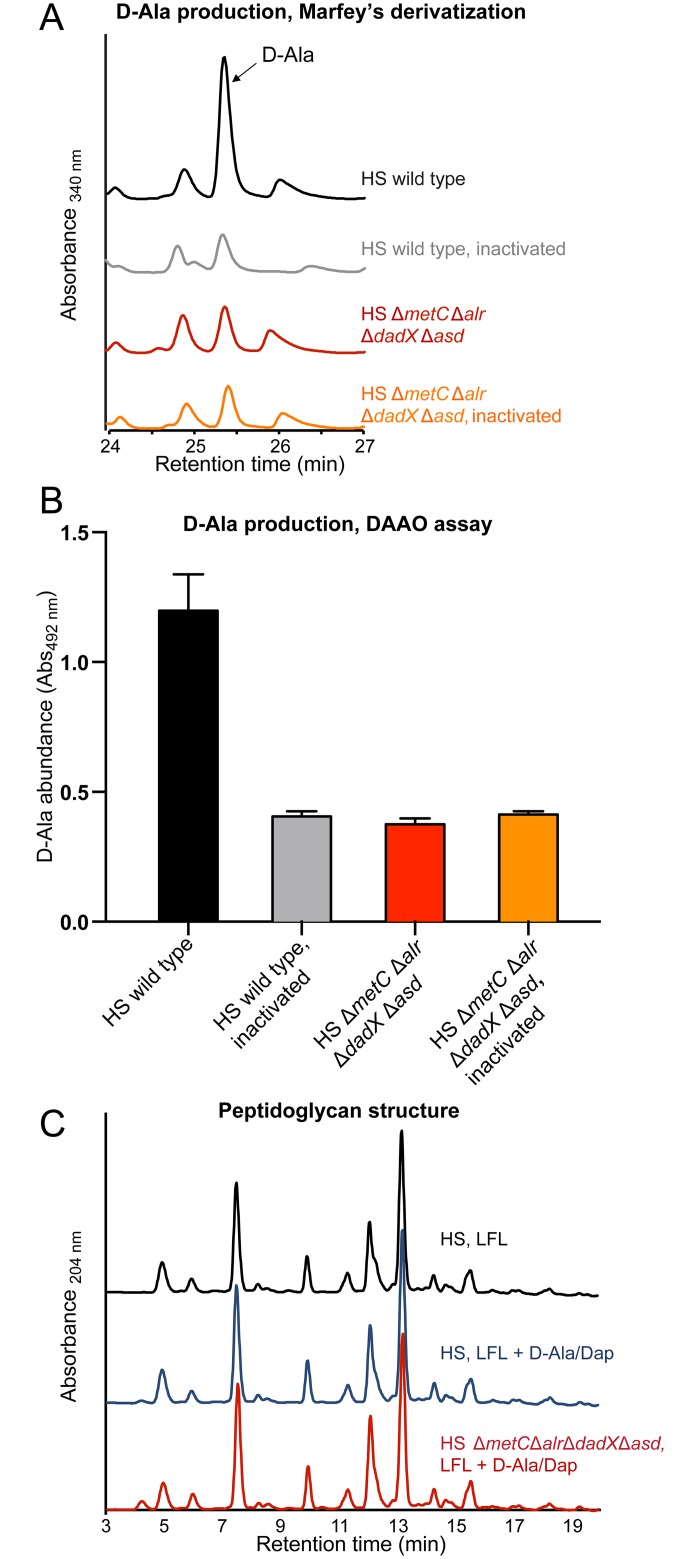
Determination of Ala-racemase activity and peptidoglycan analysis. **(A, B)**. D-Ala production by crude extracts of HS wild-type (black line/ bar) and HS Δ*metC* Δ*alr* Δ*dadX* Δ*asd* (red line/ bar) determined by **(A)** Marfey's derivatization-HPLC analysis and **(B)** D-amino acid oxidase (DAAO) assay. Heat-inactivated crude extracts of HS wild type (grey line/ bar) and HS Δ*metC* Δ*alr* Δ*dadX* Δ*asd* (orange line/ bar) served as negative controls. **(C)** UPLC peptidoglycan analysis of HS wild type grown in LFL medium with (blue line) and without (black line) supplementation with D-Ala and Dap and HS Δ*metC* Δ*alr* Δ*dadX* Δ*asd* (red line) grown in supplemented LFL. LFL: L-form-like medium. Analysis was repeated 3 times; chromatograms from one representative experiment are shown.

Despite its complete dependence on externally supplied D-Ala and Dap, HS Δ*metC* Δ*alr* Δ*dadX* Δ*asd* has a normal growth rate (compared to its parental wild-type strain as control) in appropriately supplemented medium (Fig 1E). As confirmation of this conditional phenotype we carried out biochemical cell wall analyses to evaluate if auxotrophic HS Δ*metC* Δ*alr* Δ*dadX* Δ*asd* was able to incorporate externally acquired D-Ala and Dap into a peptidoglycan of normal composition. First, we confirmed the complete absence of endogenous D-Ala racemization activity in HS Δ*metC* Δ*alr* Δ*dadX* Δ*asd*. Alanine racemase activity was quantified by measuring the production of D-Ala from L-Ala in bacterial crude extracts (see material and methods) by two different techniques: Marfey's (FDAA) derivatization and D-amino acid oxidase (DAAO) assays (Fig 2A and 2B). No residual alanine racemase activity was detectable in HS Δ*metC* Δ*alr* Δ*dadX* Δ*asd*. Second, we compared the peptidoglycan structure between laboratory-grown (D-Ala and Dap supplemented) HS Δ*metC* Δ*alr* Δ*dadX* Δ*asd* and wild type. The muropeptide profile obtained by UPLC analysis showed that the peptidoglycan structures of both strains were indistinguishable (Fig 2C). Thus, *in-vitro* grown HS Δ*metC* Δ*alr* Δ*dadX* Δ*asd*, externally supplied with D-Ala and Dap, has a cell wall of normal composition.

### Sustained bacterial activity under non-permissive conditions

**Fig 3:**
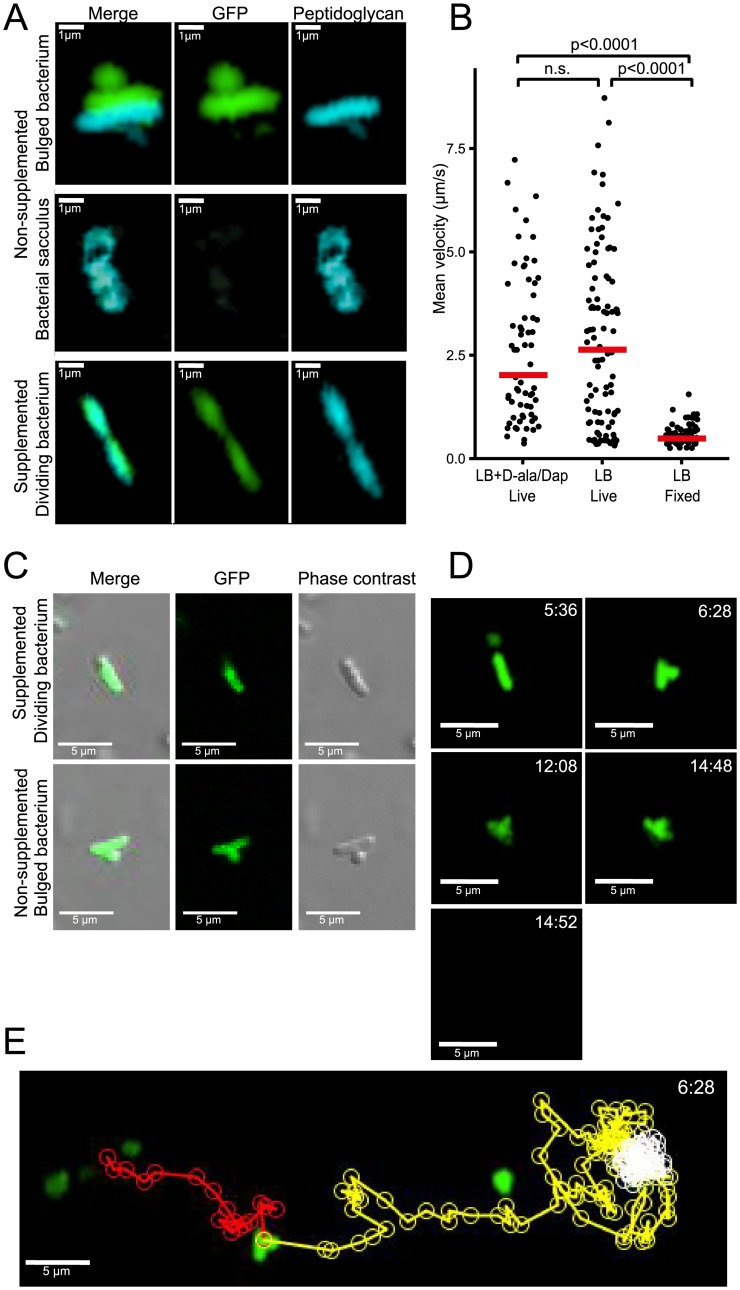
Bacterial activity and survival under non-permissive conditions. **(A)** Frame shots of a bacterium displaying cellular bulging (top), an empty bacterial sacculus after autolysis (middle), and a dividing bacterium undergoing septum formation with intact cell wall formation (bottom). Green, cytoplasmic eGFP; blue, HADA-labelled cell wall. (B-D) eGFP-expressing (green) bacteria grown in LB medium containing D-Ala and Dap were diluted in soft agar medium containing no supplements (LB, Live), D-Ala and Dap (LB + D-Ala/Dap, Live), or fixed with 4% *para*-formaldehyde (LB, Fixed) on a microscopy slide. Time-lapse videos were recorded using a 2-photon microscope and quantified with Volocity software. **(B)** Mean velocities of individual HS Δ*metC* Δ*alr* Δ*dadX* Δ*asd* under the three indicated conditions. Statistical analysis: Kruskal-Wallis test with KruskalMC as post hoc. **(C)** Frame shots of confocal eGFP overlaid with phase contrast images of a D-Ala/Dap-depleted bacterium displaying cellular bulging (bottom), and a D-Ala/Dap-supplemented control of normal morphology (top). **(D)** Frame shots of bacterium displaying cellular bulging. Top right time stamps indicate time after D-Ala/Dap depletion. **(E)** Track of the bacterium shown in panel C, before bulge formation (red path), after bulge formation (yellow path), after stopping and until lysis (white path).

We have previously shown that the initial gastrointestinal passage of D-Ala/Dap auxotrophs and wild-type *E*. *coli* is similar [6] showing that the majority of bacteria survive the intestinal passage but cannot sustain colonization without reproduction *in vivo*. D-Ala/Dap deficiency has a highly cell cycle-dependent phenotype. Whereas non-dividing cells are stable, dividing cells at initiation of binary fission undergo a programmed autolytic cell death, an active process that is linked to the cell wall rearrangements preceding binary fission [18]. Whilst autolysis itself is an activity-dependent cellular process, little is known about the impact of D-Ala/Dap-deficiency on bacterial activity prior to autolysis. We therefore used 2-photon microscopy to dynamically track the swimming velocity of D-Ala/Dap-deprived HS Δ*metC* Δ*alr* Δ*dadX* Δ*asd* (which is flagellated and motile) over time as a proxy for bacterial energy status and functional integrity of the bacterial cell envelope (into which the flagellar rotor is embedded). Tracking growth of live HS Δ*metC* Δ*alr* Δ*dadX* Δ*asd* that had been cultured in medium containing D-Ala/Dap as well as the metabolic peptidoglycan label hydroxycoumarin-carbonyl-amino-D-alanine (HADA; [19]) after transfer to D-Ala/Dap-supplemented and non-supplemented medium, respectively, we could confirm that bacteria in supplemented media were able to proliferate with intact cellular septum formation and division (Fig 3A, bottom panels; Fig 3C top). In sharp contrast non-supplemented bacteria at this stage began to display mid-lateral bulging with cytoplasm membrane protrusion due to a breach in the cell wall rigidity and the consequent loss of turgency (Fig 3A, top panels; Fig 3C bottom panels), later followed by lysis leaving behind empty peptidoglycan sacculi (Fig 3A, middle panels). To study bacterial activity prior to these processes, GFP-expressing bacteria were diluted into D-Ala/ Dap-deprived and non-deprived soft agar medium (to slow down swimming for more accurate velocity measurement) and tracked by time-lapse 2-photon microscopy over an observation period between 5 and 60 min after D-Ala/Dap depletion. We observed that HS Δ*metC* Δ*alr* Δ*dadX* Δ*asd* maintained identical mean velocities in non-supplemented medium as in supplemented control medium (Fig 3B). Although in a small proportion (6%) of tracked cells the early stages of autolysis with mid-lateral outer-membrane bulge formation (as previously described for beta-lactam antibiotic-induced autolysis in [18]; Fig 3D) could be observed, even bulge formation had no immediate impact on motility of the affected cells; bulged cells stopped swimming only approximately 3 min before cell death (sudden release of cytoplasmic GFP within <4 seconds; see example shown in S1 Video and Fig 3D and 3E), having little impact on mean velocity. These data collectively show that the activity and agility of D-Ala/ Dap-deprived HS Δ*metC* Δ*alr* Δ*dadX* Δ*asd* remains largely unaffected until immediately before autolytic cell death, closely resembling beta-lactam antibiotic-induced cell death [18].

### Transient intestinal mono-colonization

**Fig 4:**
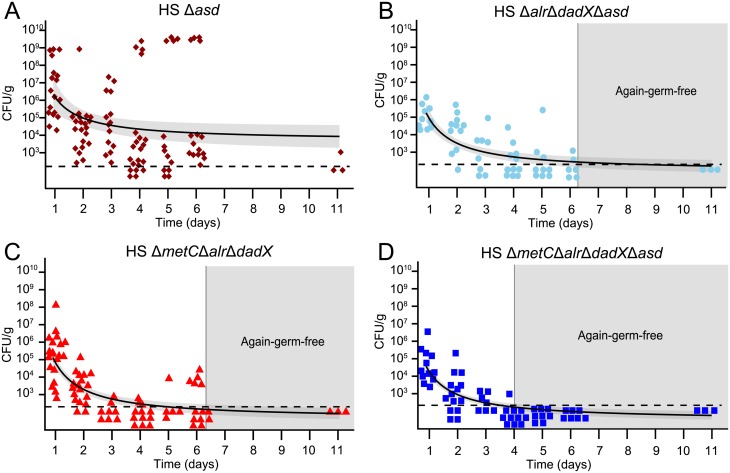
Transient intestinal colonization kinetics of auxotrophic *E*. *coli* HS. Germ-free mice were inoculated by gavage with 4.3±1.0 x 10^10^ CFU of either **(A)** HS Δ*asd* (brown symbols), **(B)** HS Δ*alr* Δ*dadX* Δ*asd* (light blue symbols), **(C)** HS Δ*metC* Δ*alr* Δ*dadX* (red symbols), or **(D)** HS Δ*metC* Δ*alr* Δ*dadX* Δ*asd* (blue symbols). Bacterial numbers in fresh feces were quantified at the indicated time points by plating on D-Ala- and Dap-supplemented LFL medium. Each symbol represents one individual. Data of three independent experiments were combined. Light-grey shaded area indicates the time point upon which all mice have regained germ-free status. Black line represents the exponential-decay-fitted curve (CFU = a1/time); the dark-grey shaded area around the curve indicated the confidence interval of the fitted curve (95% confidence); horizontal dotted line indicates the lower detection limit.

Next, we evaluated the intestinal colonization kinetics of the optimized, fully D-Ala auxotrophic strain by gavaging germ-free mice with identical doses (4.3±1.0 x 10^10^ CFU; mean±SD in 200μL PBS) of the congenic mutants HS Δ*asd* (D-Ala^+^ Dap^aux^), HS Δ*metC* Δ*alr* Δ*dadX* (D-Ala^full-aux^ Dap^+^), HS Δ*alr* Δ*dadX* Δ*asd* (D-Ala^aux^ Dap^aux^) and HS Δ*metC* Δ*alr* Δ*dadX* Δ*asd* (D-Ala^full-aux^ Dap^aux^), respectively (Fig 4). The oral-fecal passage and intestinal persistence of the 4 strains was compared over the course of 11 days by quantification of LFL-culturable bacteria from fresh feces. Dap^aux^ single-auxotroph HS Δ*asd* showed prolonged bacterial shedding until at least day 11, indicative of residual *in vivo* proliferation (Fig 4A). In one cage of 4 mice inoculated with HS Δ*asd*, spontaneous occurrence and transmission of a m-Dap auxotrophy revertant led to high-level colonization of all 4 affected individuals (Fig 4A and S1 Fig). Mice that were inoculated with either HS Δ*metC* Δ*alr* Δ*dadX* or HS Δ*alr* Δ*dadX* Δ*asd* returned to germ-free status within 3–6 days, but with highly variable and irregular kinetics (Fig 4B and 4C). In contrast, all animals inoculated with HS Δ*metC* Δ*alr* Δ*dadX* Δ*asd* consistently returned to again-germ-free status within 3–4 days (Fig 4D). No double-revertants were recovered *ex vivo* on D-Ala/ Dap-free LFL medium. These data collectively show that the additional deletion of *metC* effectively prevented the occurrence of prolonged intestinal persistence and increased robustness of reversible colonization of germ-free animals.

The early gastrointestinal transit between 5 and 9 hours post inoculation was sampled in 1-hour intervals (S2 Fig), revealing that the peak fecal bacterial densities of HS Δ*metC* Δ*alr* Δ*dadX* Δ*asd* remained within an order of magnitude as the density of the gastric inoculum (around 2x10^10^-2x10^11^ CFU/g; S2 Fig), indicating that a large fraction of the inoculated bacteria survived the intestinal passage.

### Intact IgA-stimulatory activity *in vivo*

**Fig 5:**
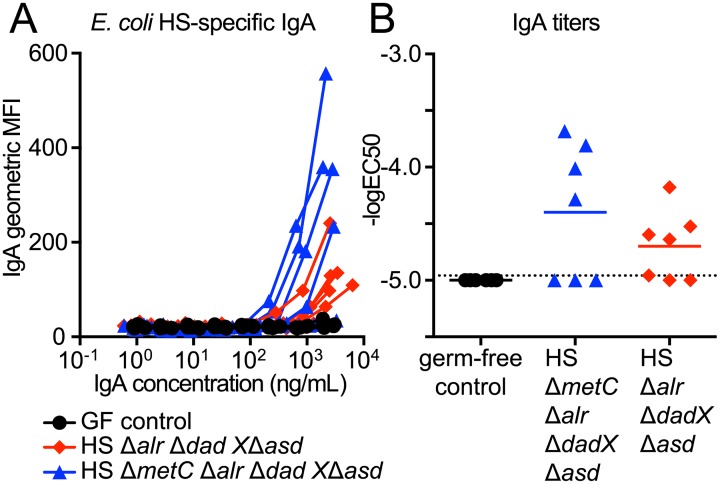
IgA stimulatory activity of transiently colonizing *E*. *coli* HS Δ*metC* Δ*alr* Δ*dadX* Δ*asd*. IgA-containing Intestinal lavages were prepared from the animals 28 days post inoculation with *E*. *coli* HS Δ*alr* Δ*dadX* Δ*asd* (red symbols) and HS Δ*alr* Δ*dadX* Δ*asd* Δ*metC* (blue symbols), and germ-free control animals (black symbols). **(A)** IgA decoration of *E*. *coli* HS incubated with varying concentrations of intestinal secretory IgA. Geometric means of IgA-fluorescence intensities (IgA geoMFI) were plotted against IgA concentration in the assay, resulting in titration curves. **(B)** Anti-live-*E*. *coli* HS IgA antibody titers, expressed as -logEC_50_, calculated by 4-parameter-curve-fitting of the data shown in (A). Dotted line, lower detection limit.

Transiently colonizing D-Ala/ Dap-auxotrophic *E*. *coli* strains were originally developed to study the dynamics and dose-response relationship of commensal bacterial induction of intestinal immunoglobulin A (IgA) [6]. In these studies we showed that the bacterial induction of IgA strongly depended on a mucosal exposure to live *E*. *coli*, and killed bacteria were highly attenuated in their IgA stimulatory potential [6]. We therefore used the induction of live-*E*. *coli* HS-specific IgA as a sensitive readout for testing if additional mutation of *metC* negatively affected the IgA stimulatory activity. We compared the intestinal IgA immunogenicity of HS Δ*metC* Δ*alr* Δ*dadX* Δ*asd* and its parental strain HS Δ*alr* Δ*dadX* Δ*asd in vivo*. The intestinal secretory IgA for this analysis was isolated from the germ-free mice presented in Fig 4B and 4D, 28 days after they had received equivalent doses of HS Δ*metC* Δ*alr* Δ*dadX* Δ*asd* and HS Δ*alr* Δ*dadX* Δ*asd*, respectively. Quantification of the anti-*E*. coli HS IgA titers in a live bacterial flow cytometry assay (see Methods section and S3 Fig for details) revealed no decrease of IgA induction by *metC* mutation (Fig 5). Thus, the described genetic optimization in *E*. *coli* HS Δ*metC* Δ*alr* Δ*dadX* Δ*asd* improved reversibility of intestinal colonization without compromising its intestinal IgA stimulatory activity.

## Discussion

Early attempts to generate *E*. *coli* strains that can no longer survive in their natural environment (primarily the lower intestinal lumen) date back to the 70s with the purpose to more effectively confine recombinant genetic material to controlled laboratory environments. One of the most prominent “safety” strains from this early era of biotechnology, *E*. *coli* K-12 Chi1776 (genotype: F^-^ Δ[*gal-uvrB*]40 Δ[*bioH-asd*]29 *supE*42 *thyA*142 *glnV*42 *hsd*R2 *cycB*2 *cycA*1 *gyrA*25 *tonA*53 *dapD*8 *minA*1 *minB*2 *rfb-2 oms-1 oms-2* λ^-^) [20] combines Dap and thymidine auxotrophies with several other debilitating mutations that make it sensitive to bile salts, UV light, and osmotic stress, resulting in an environmentally very unstable organism that does not survive the gastrointestinal transit in germ-free rats [21]. The less enfeebled Dap and thymidine auxotroph *E*. *coli* DP50 (genotype: *fhuA53 dapD*8 *lacY glnX*44 Δ*(gal-uvrB)*47 λ^-^ *tyrT*58 *gyrA*29 Δ*thyA*57 *hsdS*3) was designed to be more stable under permissive laboratory conditions. It also better survives the gastrointestinal transit, but persistently colonized germ-free animals in the form of Dap^+^ revertants, if inocula of greater than 10^7^ CFU were administered [22], similar to our HS Δ*asd* mono-colonization data in this study (see Fig 4A).

Following a similar strategy we more recently developed an *in vivo* growth-defective strain of K-12 by combining Dap auxotrophy (which alone was insufficient to reliably control germ-free intestinal colonization) with the synergistically acting auxotrophy for D-Ala to generate a fully reversibly colonizing K-12 derivative strain HA107 that could be inoculated repeatedly in doses above 10^10^ CFU without permanent intestinal colonization [6]. Although HA107 does not escape from its *in vivo* cell wall biosynthesis deficiency, it survives the gastrointestinal transit similarly well as its congenic non-auxotrophic parental strain, making it an effective tool for live bacterial conditioning of germ-free animals [6]. In the present report we further refined this approach by adapting it to the more resilient and less laboratory-adapted commensal strain *E*. *coli* HS, which required optimization of D-Ala auxotrophy.

Our data demonstrate that in *E*. *coli* HS and likely also other *Enterobacteriaceae* D-Ala is a more essential metabolite than Dap for peptidoglycan biosynthesis and growth. We continue to combine D-Ala auxotrophy with Dap auxotrophy as a “second hit” strategy, since it was still possible to select *in vitro* (but never *in vivo*) D-Ala^aux^ revertants with Δ*metC* Δ*alr* Δ*dadX* genotype that can grow (albeit poorly) without D-Ala supplementation by an unknown mechanism. Additional work will be required to identify (and prevent) this yet unknown escape pathway. An advantage of purely D-Ala auxotrophic strains would be the entirely cell-wall-specific phenotype and the universal applicability of D-Ala auxotrophy since D-Ala, in contrast to Dap, is an essential metabolite of all known Eubacteria.

A main scientific application of this model is the bacterial conditioning and concomitant “normalization” of microbially shaped body functions (such as the immune system) in germ-free animals without permanent microbial colonization. Many bacterially modulated processes depend on live microbes. We therefore deliberately did not target enterobacterial colonization factors like bile acid resistance (LPS-O-antigen), adhesion (fimbriae etc.) factors or motility that may be important for productive microbe-host interaction in the intestinal mucosa. As a consequence transiently colonizing *E*. *coli* HS retains a high IgA immunogenicity and survives the intestinal transit. Also for applications using inactivated bacterial preparations or products in biological systems *in vivo* or *in vitro* the use of this model for production of such materials effectively avoids the contamination with surviving bacteria.

In conclusion, we extended a robust transient mono-colonization model from a laboratory strain to a biologically more representative and more resilient intestinal commensal *E*. *coli* strain. This model can serve as a technology platform for numerous scientific applications. It may be used as a “sterile” biological vector for proteins, metabolites or signaling molecules that need to be delivered directly *in situ* or cannot be stably purified. More generally, it represents a live bacterial conditioning system for axenic animals or other sterile biological systems for the detailed study of host-microbial interactions. Many other future applications are thinkable, and the genetic approach may be extended to other microbial species.

## Materials and Methods

### Animal colonization experiments

Germ-free animals were re-derived from C57BL/6 mice and maintained germ-free in flexible film isolators in the Genaxen Foundation Clean Mouse Facility (CMF) of the University of Bern as described [23]. Experimental germ-free mice were aseptically transferred to autoclaved sealsafe-plus IVCs under positive pressure (Tecniplast, Italy) in a barrier unit of the Genaxen Clean Mouse Facility. Cage changes were carried out under strictly aseptic conditions. In all experiments animals were provided with sterile mouse chow (Kliba 3437; autoclaved) and autoclaved water ad libitum. All experiments were performed according to protocols approved by the Bernese Cantonal Ethical committee for animal experiments and carried out in accordance with Swiss Federal law for animal experimentation (license number BE91/14).

To generate contamination-free bacterial inoculums, D-Ala (200 μg/μ)- and Dap (50 μg/mL)-supplemented autoclaved LB medium in sterile-filter-sealed flasks, was aseptically inoculated from single colonies of the test bacterium and incubated shaking at 150 rpm at 37°C for 16 hours. Bacteria were harvested by centrifugation (10 min, 4816 x g, 4°C) in a sterile aerosol-proof assembly, washed in autoclaved sterile PBS and concentrated to a density of 2 x 10^11^ CFU/mL in sterile PBS, performed aseptically under a sterile laminar airflow. The bacterial suspensions were aseptically aliquoted in autoclaved plastic tubes and sealed in a sterilized secondary containment. The sterile tubes containing the inocula and germ-free mice were aseptically imported into a sterilized laminar flow hood laid out with sterile surgical drapes, and each animal inoculated with 200 μL of bacterial suspension (containing 4 x 10^10^ CFU in sterile PBS, at a density of 2 x 10^11^ CFU/mL) by gavage, carried out wearing sterile surgical gowns and sterile surgical gloves. Fresh fecal pellets were collected aseptically, suspended in sterile PBS, and plated in serial dilutions on D-Ala/ Dap-supplemented or non-supplemented LFL agar and incubated aerobically at 37°C for ≥ 24 hours.

### Bacterial culture

LB medium (Sigma-Aldrich) was used as the standard growth media. Where required, the following supplements were added to the media: ampicillin (Sigma, 100 μg/mL), tetracycline (Sigma, 12.5 μg/mL), kanamycin (Sigma, 50 μg/mL), meso-diaminopimelic acid (Sigma, 50 μg/mL), D-alanine (Sigma, 200 μg/mL). L-form-like media (LFL) was prepared in two parts: 75.2 g/L brain-heart infusion broth, 20 g/L agar; and separately 10 mM MgSO_4_, 200 g/L, sucrose, and mixed in equal parts after autoclaving. The frequencies of auxotrophy revertants were measured by plating stationary phase bacterial culture on LB or LFL agar plates containing D-Ala+Dap, D-Ala only, Dap only, or no supplements and incubated at 37°C. Revertant frequencies are equivalent to the ratio revertant CFU/ total CFU.

### Bacterial genetic engineering

All bacterial strains used or generated in this study are specified in S1 Table. The *E*. *coli* HS wild type strain was kindly provided by Jim Nataro from the University of Virginia School of Medicine, Charlottesville VA, USA and is a replicate of the same bacterial stock that was fully sequenced by Rasko *et al*. [14] (GenBank accession no. CP000802). All deletions were carried out by Lambda Red recombineering. Mutagenesis primer sequences are specified in S2 Table. (i) Strain HA126 (Δ*asd*::*tetRA*) was generated by deletion of *asd* using recombineering plasmid pKD46 as described [24] with minor modifications: a *tetRA* recombineering amplicon was amplified from genomic *tetRA* template DNA (isolated from a Tn10-containing bacterial strain) with primers HS-asd-mutF and HS-asd-mutR, and 2 mM L-arabinose added to express recombinase for 1 hour before the culture was stopped. (ii), Following the same protocol, *alr* in HA126 was deleted using recombinase plasmid pKD46 and a *flp-kan-flp* recombineering cassette amplified with primers HS-alr-mutF and HS-alr-mutR from template plasmid pKD4, followed by elimination of the *kan* resistance gene using FLP recombinase plasmid pCP20 as previously described [25], resulting in strain HA130 (Δ*alr*::*flp* Δ*asd*::*tetRA)*; this procedure leaves behind one *flp* site). (iii), following the same procedure, *dadX* was deleted in HA 130, using primers HS-dadX-mut-F and HS-dadX-mutR, to generate strain HA132 (Δ*alr*::*flp* Δ*dadX*::*flp-kan-flp* Δ*asd*::*tetRA*). (iv), the *kan* resistance was removed from HA132 using pCP20, and *metC* was deleted using the heat-shock regulated recombinase expression plasmid pSIM6, primers HS-metC-mutF and HS-metC-mutR, and the *flp-kan-flp* template plasmid pKD4, following a recently published protocol [26], resulting in strain HA416 (Δ*metC*::*flp-kan-flp* Δ*alr*::*flp* Δ*dadX*::*flp* Δ*asd*::*tetRA*). (v), To generate strain HA417 (Δ*metC*::*flp-kan-flp* Δ*alr*::*flp* Δ*dadX*::*flp*), the Δ*asd*::*tetRA* allele in HA416 was replaced with the wild-type *asd* allele, using pSIM6 in combination with a recombineering amplicon produced with primers asd_F and asd_B and wild type genomic template DNA. HA417 was isolated by positive selection of recombinants on LB containing D-Ala. All deletions were verified phenotypically and by control PCR (control primers specified in S2 Table). Plasmids (see S1 Table for a complete list) were introduced by electroporation following standard protocols.

### Insertion element identification

The genomic metJ region of E. coli HS was amplified by PCR using primers metJ_F and metJ_B (S2 Table), and Sanger sequenced (Microsynth, TWON, Switzerland) using the same primers. The obtained sequences were compared using BLAST with the nr database and the best match was considered to be the correct.

### Two- photon and confocal microscopy

A subculture of HS Δ*metC* Δ*alr* Δ*dadX* Δ*asd* harboring eGFP expression plasmid pM979 was grown in LB containing D-Ala, Dap, ampicillin, 50 nM HADA, [27] for approximately 3 hours to reach OD_600_ = 0.6 and cooled down on ice. Small aliquots of this culture were sedimented by centrifugation and re-suspended 1:100 in fresh medium containing ampicillin, and either (i) no further chemicals, (ii) D-Ala and Dap, or (III) 4% *para*-formaldehyde (end concentration), and poured on a microscopy slide with matching media covered with low-melting-point 0.1% agarose. Time lapse videos were recorded immediately using an Olympus BX50WI fluorescence 15 microscope (three z-stacks with 3 μm spacing, 150 μm square sections, fast mode) attached to a 2-PM scanner (TrimScope system, LaVision Biotec, Bielefeld, Germany) equipped with a 20X objective (numerical aperture = 0.95) and heated stage. Image sequences were transformed into volume-rendered four-dimensional movies using Volocity software (Improvision), which was also used for semi-automated tracking of bacteria motility. From the acquired videos 85 (supplemented) or 100 (non-supplemented and PFA) bacterial tracks were selected based on duration (≥10 consecutive time points, 3.3 frames/s), size (≥3 μm diameter) and the intensity of the objects (more than 1900 voxels). These tracks were imported into R and processed into plots using ggplot2 and dplyr.

For confocal microscopy, identically prepared bacterial preparations were imaged with a Zeiss LSM 710 confocal laser-scanning microscope, time-lapse movies of GFP and Phase contrast (three z-stacks with 3 μm spacing, 150 μm square sections, fast mode channels) were recorded simultaneously using a beam splitter with a 40X oil objective (numerical aperture = 1.3) and heated stage. Image sequences were transformed using Imaris software.

### Enzyme-linked immunosorbent assay (ELISA) for IgA

Total concentrations of IgA in mouse intestinal lavage were determined by sandwich ELISA. Coating antibodies were goat anti-mouse IgA (Southern Biotech, 1040–01); detection antibodies were horseradish peroxidase (HRP)–conjugated goat-anti-mouse IgA (A3673, Sigma). Purified monoclonal isotype control IgA (Becton Dickinson, clone M18-254, 553476) served as standard.

### Live bacterial flow cytometry and IgA response quantification

Live bacterial flow cytometry quantification of *E*. *coli* HS-specific Immunoglobulin (Ig)A titers (expressed as—EC_50_ values) were carried out previously described [6]. Briefly, *E*. *coli* HS was grown in 0.2 μm membrane-filtered LB broth overnight at 37°C without shaking. 1 mL of culture was gently pelleted for 3 min at 4816 x g in a Heraus Fresco 21 centrifuge and washed 3 times with sterile-filtered 2% BSA/ 0.005% NaN_3_/ PBS before re-suspending at a density of approximately 10^7^ bacteria/mL. Intestinal IgA lavages were collected by rinsing the small intestine with 5 mL of 1% soybean-trypsin-inhibitor/ 0.05 M EDTA/ PBS. The intestinal washes were then centrifuged at 4816×g, 20 min; the supernatant sterile-filtered to remove bacteria-sized particles, and serially diluted in sterile-filtered 2% BSA/ 0.005% NaN_3_/ PBS. Serially diluted IgA-solution and bacterial suspension were mixed 1:1 and incubated at 4°C for 1 h. Bacteria were washed twice in sterile-filtered 2% BSA/ 0.005% NaN_3_/ PBS before re-suspending in monoclonal FITC-anti-mouse IgA (clone 10.3; Becton Dickinson). After 1 h incubation at 4°C the bacteria were washed twice with sterile-filtered 2% BSA/ 0.005% NaN_3_/ PBS and re-suspended in 2% *para*-formaldehyde/ PBS for acquisition on a FACSArray SORP flow cytometer (Becton Dickinson) using FSc and SSc parameters in logarithmic mode. GeoMean fluorescence intensities were plotted against IgA concentrations analyzed using FlowJo software (Treestar, USA) and Graphpad prism software 4-parameter curve fitting to calculate—EC50 titers as previously described [6] and summarized in S3 Fig.

### Isolation of peptidoglycan and UPLC analysis

To isolate murein sacculi, cultures were pelleted, resuspended in 2 mL of medium and slowly added to 2 mL of boiling 10% SDS while stirring. After boiling for 2 h, they were stirred overnight at room temperature. Cell wall material was then pelleted by ultracentrifugation (60’000 rpm, 10 min) and washed with purified water to remove SDS. Samples were digested with pronase E (100 μg/mL) in 10 mM Tris-HCl, pH 7.5, 1 h at 60°C to remove Braun’s lipoprotein. After addition of SDS to a final concentration of 1% (w/v), reactions were heat-inactivated and detergent was removed by washing in MQ water. Purified peptidoglycan was re-suspended in 100 μL of 50 mM NaPO_4_ buffer pH 4.9 and treated with 100 μg/mL muramidase (Cellosyl) for 16 h at 37°C. Muramidase digestion was stopped by boiling and coagulated proteins were removed by 10 min centrifugation at 14’000 rpm. Supernatants were reduced by adding sodium borate pH 9.5 and sodium borohydride to a final concentration of 10 mg/mL and incubating at RT for 30 min. Finally, samples were adjusted to pH 3.5 with phosphoric acid. Muropeptides were separated in a 20-min linear gradient of 50 mM NaPO_4_, pH 4.35, to 50 mM NaPO_4_, pH 4.95, and 15% (v/v) methanol on an AQUITY ultra-performance liquid chromatography (UPLC) BEHC18 column (130 Å, 1.7 μm, 2.1 mm × 150 mm; Waters, USA), and detected by absorption at wavelength 204 nm.

### Alanine racemase activity assay

Cleared cell lysates were prepared from cultures and assayed for alanine racemase activity. Lysates were prepared from cultures grown in LB supplemented with D-Ala and Dap (OD600 was measured for normalizing the number of cells in each sample). Cells were collected by centrifugation and then washed twice with ice-cold HEPES 50 mM pH 7.5. By three passages through a French press re-suspended cells were disrupted and then lysates were cleared of cell debris and membranes by centrifugation at 20’000 rpm for 30 min at 4°C. Alanine racemization assays (adapted from [28,29]) were performed in 1.5 mL Eppendorf tubes in a total volume of 200 μL by adding 160 μL of the soluble fraction of crude extract, L-Ala and pyridoxal phosphate (PLP) at 50 mM and 20 μM respectively (final concentrations). After incubation at 37°C for 45 min the reaction was quenched by adding 40 μl of 2 M HCl.

L-FDAA (1-fluoro-2-4-dinitrophenyl-5-L-alanine amide, Marfey’s reagent, Thermo Scientific) was used for the derivatization of amino acids [28,30]. First quenched enzyme reactions were neutralized with 40 μL of 2 M NaOH and then a 50 μL aliquot of the sample was mixed with 100 μL Marfey's reagent (0.5% solution in acetone) and 20 μL 1 M NaHCO_3_. The derivatization mixture was incubated at 80°C for 10 min and the reaction was stopped with 2 M HCl. After cooling down to room temperature samples were diluted with 200 μL of a mixture 9:1 of buffers A (triethylamine-phosphate 50 mM pH 3) and B (triethylamine-phosphate 50 mM pH3, 40% acetonitrile). Samples were filtered and 100 μL were injected in the HPLC (high-pressure liquid chromatography). Amino acids were separated in a 45 min linear gradient of triethylamine phosphate/acetonitrile with an Aeris peptide column (250 x 4.6 mm; 3.6 μm particle size, Phenomenex, USA) and detected at Abs. 340 nm. D-Ala and L-Ala were used as standards of to establish retention times.

DAAO reaction coupled to peroxidase and 2,3-diaminophenazine was performed for detection of the D-Ala produced by the *in vitro* as previously described [31]. The formation of the colorimetric product was measured at 492 nm.

### Data analysis and statistical analysis

All data analysis was done using the R 3.1.1 statistical program [32] and the ggplot2 [33], dplyr [34] and pgirmess [35] packages. All scripts can be downloaded from https://github.com/cuencam/HA416, and data are available on request. Statistical tests are specified in the figure legends.

## Supporting Information

S1 Fig Selection of spontaneous *asd* auxotrophy revertants in *E*. *coli* HS Δ*asd*-inoculated animals. 4 Germ-free mice (also depicted in Fig 4A) were inoculated by gavage with approximately 4x10^10^ CFU of HS Δ*asd*. **(A)** CFU counts from each mouse over time, each individual highlighted in a different color. **(B)** PCR amplification of the genomic *asd* region of HS wild-type (lane 1), HS Δ*asd* (exact genotype: Δ*asd*::*tetRA*; longer PCR fragment verifies allelic exchange of *asd* by *tetRA* cassette) original stock (lane 2), and HS Δ*asd* re-isolate from mouse 3 (verifying the correct genotype of this revertant), verifying colonization with a revertant clone of the correct inoculated. Lane L contains molecular ladder. **(C, D)** Colony morphology of 4 revertant clones re-isolated from mouse 3 on day 2 (clone 1) and day 3 (clone 2), mouse 1 on day 3 (clone 3), and mouse 2 on day 3 (clone 4) on supplemented **(C)** and unsupplemented **(D)** LFL agar plates.
(PDF)

S2 Fig Early intestinal colonization kinetics of auxotrophic *E*. *coli* HS. Early time points of the experiment presented in main Fig 4 are shown. Germ-free mice were inoculated by gavage with around 4x10^10^ CFU of either **(A)** HS Δ*asd* (brown symbols), **(B)** HS Δ*alr* Δ*dadX* Δ*asd* (light blue symbols), **(C)** HS Δ*metC* Δ*alr* Δ*dadX* (red symbols), or **(D)** HS Δ*metC* Δ*alr* Δ*dadX* Δ*asd* (blue symbols). Each symbol represents one individual; data are combined from three independent experiments. Black line represents the exponential-decay-fitted curve (CFU = a1/time) with the 95% confidence interval shown as dark-grey shaded area. The vertical gray line marks the time point at which all individuals have reached fecal bacterial densities 100-fold below the mean inoculum density (from top of light gray area).
(PDF)

S3 Fig Live bacterial FACS analysis and titer calculations. IgA-stained bacteria were analyzed using a BD FACSArray SORP and acquired data were exported to Treestar FlowJo. **(A)** Gating procedure: Single bacteria were defined as forward-scatter-width-(FSC-W)-low events. Forward scatter area (FSC-A) and Side scatter area (SSC-A) were used to eliminate electrical noise, bubbles and debris from the analysis. Gating Red (APC channel)-low events allowed to reduce unspecific fluorescence. Three serial 3-fold dilutions of a representative positive sample are shown. **(B)** Three representative histograms of FITC-anti-IgA resulting from 3 serial dilutions and their overlay are shown. **(C)** Titration curves shown in main Fig 5A. Geometric mean fluorescent intensities (geoMFI; accounting for the Log Normal distribution of fluorescence data) of IgA bacterial FACS staining (y-axis) was plotted against IgA concentration in the assay (x-axis) (determined by isotype-specific sandwich ELISA). **(D)** 4-parameter curve fitting of the data shown panel C and main Fig 5A. Graphpad Prism 6 software was used to fit 4-parameter logistic curves to the data. Equation: Y = Bottom + (Top- Bottom)/ (1+10^((LogEC50-X)*HillSlope)). **(E)**–LogEC50 IgA titers. The LogEC50 values were extracted from the curve parameters, which when anti-logged corresponds to the concentration of IgA required to give half-maximum IgA binding. The—LogEC50 titer thus corresponds to the Log(1/[IgA]_giving 50% binding_) the dotted line to the lower detection limit.
(PDF)

S1 Table Bacterial strains and plasmids. (DOCX)

S2 Table Primers used in this study. (DOCX)

S1 Video Representative example of a swimming bacterium undergoing bulging and autolysis. The red circle highlights the bacterium of interest; white arrow indicates the moment at which the cells starts bulging. After approximately 7 min the bacterium stops active movement, followed 3 min later by autolysis, causing an instant drop of cytoplasmatic GFP signal as it is released into the extracellular medium. The video is part of the data shown in Fig 4.
(MOV)
