# Supplementary figures and images for: D-Alanine-Controlled Transient Intestinal Mono-Colonization with Non-Laboratory-Adapted Commensal E. coli Strain HS
Source: PLoS One. 2016 Mar 22;11(3):e0151872. doi: 10.1371/journal.pone.0151872 (PMC4803232; doi:10.1371/journal.pone.0151872)

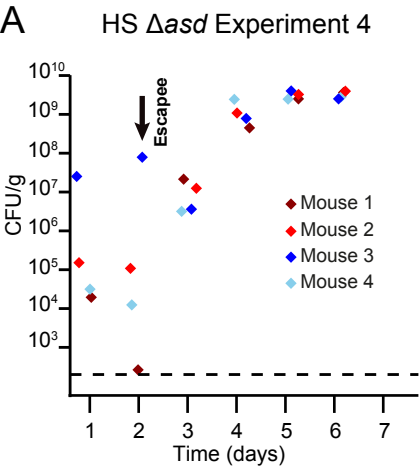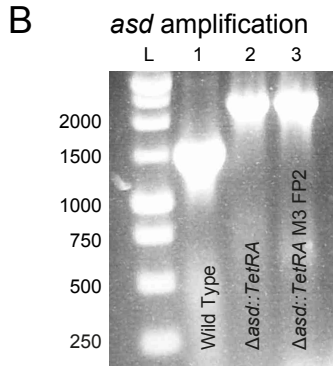

**C** LFL+ supplements

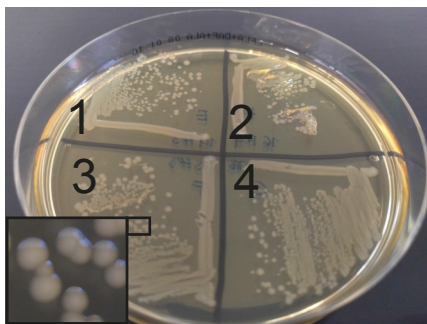

**D** LFL

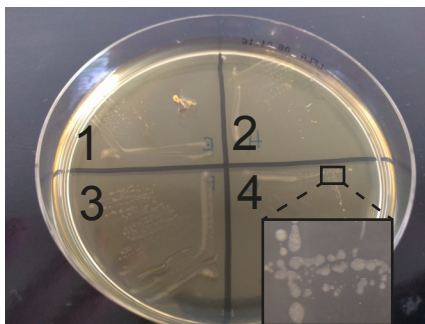

Supplement: S1 Fig — 4 Germ-free mice (also depicted in Fig 4A) were inoculated by gavage with approximately 4x10^10^ CFU of HS Δasd. (A) CFU counts from each mouse over time, each individual highlighted in a different color. (B) PCR amplification of the genomic asd region of HS wild-type (lane 1), HS Δasd (exact genotype: Δasd::tetRA; longer PCR fragment verifies allelic exchange of asd by tetRA cassette) original stock (lane 2), and HS Δasd re-isolate from mouse 3 (verifying the correct genotype of this revertant), verifying colonization with a revertant clone of the correct inoculated. Lane L contains molecular ladder. (C, D) Colony morphology of 4 revertant clones re-isolated from mouse 3 on day 2 (clone 1) and day 3 (clone 2), mouse 1 on day 3 (clone 3), and mouse 2 on day 3 (clone 4) on supplemented (C) and unsupplemented (D) LFL agar plates. (PDF) [file pone.0151872.s001.pdf]

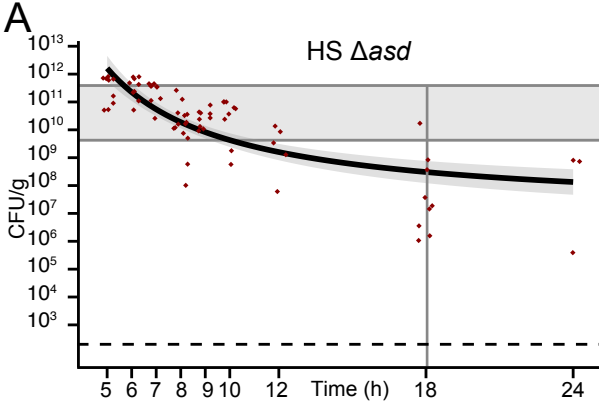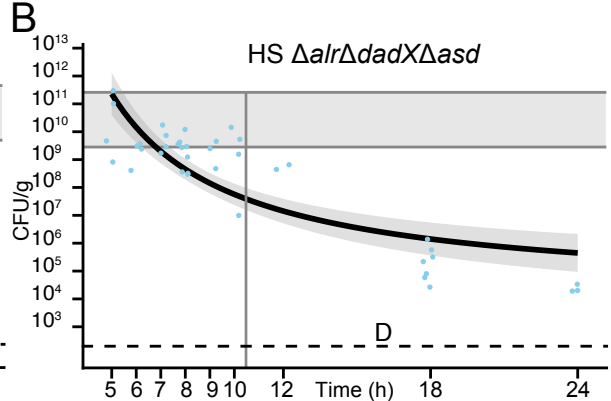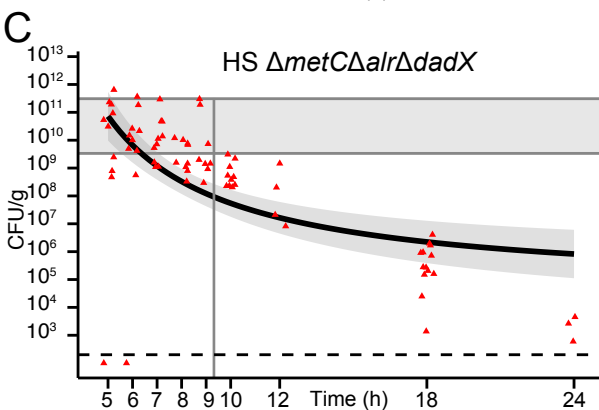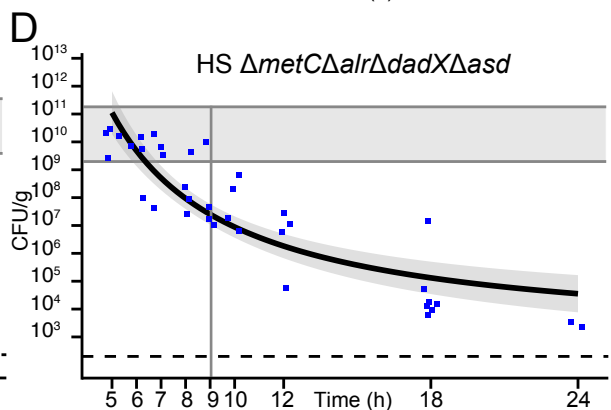

Supplement: S2 Fig — Early time points of the experiment presented in main Fig 4 are shown. Germ-free mice were inoculated by gavage with around 4x10^10^ CFU of either (A) HS Δasd (brown symbols), (B) HS Δalr ΔdadX Δasd (light blue symbols), (C) HS ΔmetC Δalr ΔdadX (red symbols), or (D) HS ΔmetC Δalr ΔdadX Δasd (blue symbols). Each symbol represents one individual; data are combined from three independent experiments. Black line represents the exponential-decay-fitted curve (CFU = a1/time) with the 95% confidence interval shown as dark-grey shaded area. The vertical gray line marks the time point at which all individuals have reached fecal bacterial densities 100-fold below the mean inoculum density (from top of light gray area). (PDF) [file pone.0151872.s002.pdf]

1:9

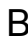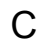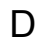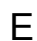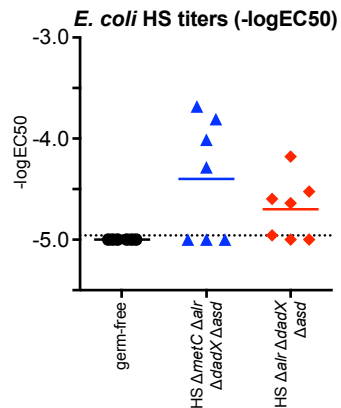

Supplement: S3 Fig — IgA-stained bacteria were analyzed using a BD FACSArray SORP and acquired data were exported to Treestar FlowJo. (A) Gating procedure: Single bacteria were defined as forward-scatter-width-(FSC-W)-low events. Forward scatter area (FSC-A) and Side scatter area (SSC-A) were used to eliminate electrical noise, bubbles and debris from the analysis. Gating Red (APC channel)-low events allowed to reduce unspecific fluorescence. Three serial 3-fold dilutions of a representative positive sample are shown. (B) Three representative histograms of FITC-anti-IgA resulting from 3 serial dilutions and their overlay are shown. (C) Titration curves shown in main Fig 5A. Geometric mean fluorescent intensities (geoMFI; accounting for the Log Normal distribution of fluorescence data) of IgA bacterial FACS staining (y-axis) was plotted against IgA concentration in the assay (x-axis) (determined by isotype-specific sandwich ELISA). (D) 4-parameter curve fitting of the data shown panel C and main Fig 5A. Graphpad Prism 6 software was used to fit 4-parameter logistic curves to the data. Equation: Y = Bottom + (Top- Bottom)/ (1+10^((LogEC50-X)*HillSlope)). (E)–LogEC50 IgA titers. The LogEC50 values were extracted from the curve parameters, which when anti-logged corresponds to the concentration of IgA required to give half-maximum IgA binding. The—LogEC50 titer thus corresponds to the Log(1/[IgA]giving 50% binding) the dotted line to the lower detection limit. (PDF) [file pone.0151872.s003.pdf]
