# Supplementary material for: D-Alanine-Controlled Transient Intestinal Mono-Colonization with Non-Laboratory-Adapted Commensal E. coli Strain HS
Source: PLoS One. 2016 Mar 22;11(3):e0151872. doi: 10.1371/journal.pone.0151872 (PMC4803232; doi:10.1371/journal.pone.0151872)
Supplement: S1 Table — (DOCX) [file pone.0151872.s004.docx]

| Strain/ Plasmid | Relevant genotype/phenotypes/description | Source, Reference |
| --- | --- | --- |
| *E. coli* HS | Wild type | Jim Nataro^a^, [13,14] |
| HA126 | Δ*asd::tetRA*; Tet^R^ | This study |
| HA130 | Δ*alr::flp* Δ*asd::tetRA*; Tet^R^ | This study |
| HA132 | Δ*alr::flp* Δ*dadX::flp-kan-flp* Δ*asd::tetRA*; Kan^R^ Tet^R^ | This study |
| HA416 | Δ*metC::flp-kan-flp* Δ*alr::flp* Δ*dadX::flp* Δ*asd::tetRA*; Kan^R^ Tet^R^ | This study |
| HA417 | Δ*alr::flp* Δ*dadX::flp* Δ*metC::flp-kan-flp*; Kan^R^ | This study |
| pSIM6 | Lambda red expression plasmid, Amp^R^ | [36] |
| pKD46 | Lambda red expression plasmid, Amp^R^ | [24] |
| pCP20 | Recombinase plasmid FLP^+^, λcI857^+^, λρ_R_ Rep^ts^, Amp^R^ | [25] |
| pM979 | Constitutive GFP-expression plasmid, Amp^R^ | W.-D. Hardt^b^, [37] |

^a^Department of Pediatrics, University of Virginia School of Medicine ; Charlottesville VA, USA

^b^ETH Zurich, Institute for microbiology, Zurich, Switzerlend

**Additional References:**

36. Datta S, Costantino N, Court DL. A set of recombineering plasmids for gram-negative bacteria. Gene. 2006;379: 109–115. doi:10.1016/j.gene.2006.04.018

37. Stecher B, Hapfelmeier S, Müller C, Kremer M, Stallmach T, Hardt W-D. Flagella and chemotaxis are required for efficient induction of Salmonella enterica serovar Typhimurium colitis in streptomycin-pretreated mice. Infect Immun. 2004;72: 4138–4150. doi:10.1128/IAI.72.7.4138-4150.2004
