# Supplementary material for: D-Alanine-Controlled Transient Intestinal Mono-Colonization with Non-Laboratory-Adapted Commensal E. coli Strain HS
Source: PLoS One. 2016 Mar 22;11(3):e0151872. doi: 10.1371/journal.pone.0151872 (PMC4803232; doi:10.1371/journal.pone.0151872)
Supplement: S2 Table — (DOCX) [file pone.0151872.s005.docx]

| Name | Description | Sequence |
| --- | --- | --- |
| HS*-asd*-mutF | *asd* *tetRA* cassette construction forward | ACA TTT ATA CAG CAC ACA TCT TTG CAG GAA  AAA AAC GCT TTT AAG ACC CAC TTT CAC ATT |
| HS-*asd*-mutR | *asd* *tetRA* cassette construction reverse | AGG GGC GGC ATC GCG CCC CAG ATT TAA TGA  ATA AAG ATT ACT AAG CAC TTG TCT CCT G |
| HS-*alr*-mutF | *alr* *kan* cassette construction forward | GAA TTA GGT AAT TAA AGC AAA CAC TTA TCA  AGG AAC ACA AGT GTA GGC TGG AGC TGC TTC |
| HS-*alr*-mutR | *alr kan* cassette construction reverse | ACG CCG CAT CCG GCA CAG ACA ATC AAA TAT TAC AGA ACG AGA TAT GAA TAT CCT CCT TA |
| HS-*dadX*-mutF | *dadX kan* cassette construction forward | TCC GGG CCA TTT ACA TGG CGC ACA CAG CTA  AG AAA GA GGT GTA GGC TGG AGC TGC TTC |
| HS-*dadX*-mutR | *dadX kan* cassette construction reverse | GCA CCC AGA AGA CGT TGC CTC CGA TCC GGC TTA CAA CAA GCA TAT GAA TAT CCT CCT TA |
| HS-*metC*-mutF | *metC kan* cassette construction forward | TAG TTT AGA CAT CCA GAC GTA TAA AAA CAG GAA TCC CGA CGT GTA GGC TGG AGC TGC TTC |
| HS-*metC*-mutR | *metC kan* cassette construction reverse | AAT AAA ATG TCT GCA AAA TTG TCC AAA AGT GGC AAT GTT ACA TAT GAA TAT CCT CCT TA |
| *asd*_F | Forward *asd* control primer | GCG TGC TAA CAA AGC AGG AT |
| *asd*_R | Reverse *asd* control primer | TCC CGG TAA ATC ATG AAA CA |
| *alr*2_F | Forward *alr* control primer | GAC GGT ACG CCT GAC CTT TA |
| *alr*2_R | Reverse *alr* control primer | GCG ATG GTT CTC CAG GTT TA |
| *dadX*2_F | Forward *dadX* control primer | GTT TCG ATA ACC GCA TTC GT |
| *dadX*2_R | Reverse *dadX* control primer | GCG ATG GTT CTC CAG GTT TA |
| *metC_*F | Forward *metC* control primer | AGGCGACGCTTCTGATTGAA |
| *metC_*B | Reverse *metC* control primer | GCCATGGACTTTCCTGTGGA |
| *metJ_*F | Forward *metJ* control primer | ATCCGGCCTACAAGTTCGTG |
| *metJ_*B | Reverse *metJ* control primer | TGTCGGTGAAATGTCAGGCA |
